# Quantifying interprovincial health and poverty disparities in Afghanistan: a secondary analysis of national survey and administrative data

**DOI:** 10.1080/16549716.2026.2751910

**Published:** 2026-10-23

**Authors:** Sayed Ataullah Saeedzai, Kanishka Ahmadyar, Mohammad Sediq Hazratzai, Alison M. El Ayadi

**Affiliations:** a Institute for Global Health Sciences, University of California, San Francisco, CA, USA; b Monitoring and Evaluation Department, Moraa Educational Complex, Kabul, Afghanistan; c Center for Immigrant and Refugee Health, Public Health Institute, Oakland, CA, USA; d Department of Obstetrics, Gynecology and Reproductive Sciences, University of California, San Francisco, CA, USA

**Keywords:** Health equity, Afghanistan, subnational disparities, maternal and child health, universal health coverage

## Abstract

**Background:**

Afghanistan has made national gains in maternal and child health since 2001; however, national aggregate figures may mask important subnational disparities.

**Objective:**

This study quantifies interprovincial disparities in health outcomes, service coverage, and multidimensional poverty across Afghanistan’s 34 provinces to identify areas experiencing the greatest cumulative disadvantage.

**Methods:**

We conducted a cross-sectional, province-level comparative analysis that integrated three nationally representative secondary data sources: the Afghanistan Multiple Indicator Cluster Survey (MICS) 2022–2023, the 2017 NSIA/MoPH geographic access-to-care report, and the Afghanistan Multidimensional Poverty Index. Nine indicators spanning child health outcomes, service coverage, access to care, and socioeconomic deprivation were scored on a quartile scale (1 = worst, 4 = best).

**Results:**

Composite scores ranged from 1.0 in Nuristan to 4.0 in Kabul, demonstrating marked geographic disparities. Nuristan recorded the lowest performance nationally, driven by the highest under-five mortality rate (120 per 1,000 live births), minimal skilled birth attendance (9.5%), and limited access to improved drinking water (16.3%). Zabul (1.2), Helmand (1.2), and Badghis (1.4) showed similarly compounded disadvantage, while Kabul, Panjshir (3.6), and Bamyan (3.4) ranked highest. Disadvantage clustered in southern and western provinces and was strongly associated with higher multidimensional poverty, illustrating the overlap between socioeconomic deprivation and poor health outcomes.

**Conclusions:**

National improvements in Afghanistan’s health indicators obscure severe, geographically clustered subnational disparities. Provinces with the lowest composite scores require prioritized, targeted investment in the health workforce, infrastructure, and community-based service delivery, alongside multisectoral poverty reduction, to advance Universal Health Coverage and Afghanistan’s human rights obligations.

## Paper Context

- **Main findings:** Composite health and poverty performance scores varied more than fourfold across Afghanistan’s 34 provinces, with Nuristan and several southern and western provinces facing severe, compounding disadvantage across child health, maternal service coverage, access to care, and poverty, while Kabul and a small number of central and northern provinces performed comparatively well.
- **Added knowledge:** This study is among the first to combine nine indicators spanning health outcomes, service coverage, access to care, and multidimensional poverty into a single province-level composite performance score for Afghanistan, using the most recent nationally representative data sources.
- **Global health impact for policy and action:** These findings can help the Ministry of Public Health, donors, and international partners target investment toward the most disadvantaged provinces, rather than relying on national averages that obscure these disparities.

## Background

Grounded in the 1948 Universal Declaration of Human Rights and the 1966 International Covenant on Economic, Social, and Cultural Rights, the right to health affirms that every individual is entitled to the highest attainable standard of physical and mental health [1,2]. Governments are obligated to take deliberate, progressive, and equitable steps to ensure that all people can access the services, resources, and conditions necessary to achieve this standard. These conditions extend beyond healthcare services alone and include the broader social determinants of health, such as adequate nutrition, safe housing, education, and a clean environment. Afghanistan, as a signatory to both instruments, has formally committed to upholding and progressively realizing these fundamental rights, including the right to health, within its national policies and development frameworks [3,4].

Health equity is a fundamental component of the right to health, and is operationalized through universal health coverage (UHC), which seeks to ensure that all individuals receive the health services they need without suffering financial hardship [5,6]. Achieving UHC, therefore, requires reducing avoidable and unjust disparities arising from geographic, socioeconomic, gender, ethnic, and other structural inequalities. As a central target of Sustainable Development Goal (SDG) 3 on good health and well-being [6], UHC integrates service coverage, financial protection, and quality of care.

Despite its global commitments, achieving health equity and UHC in Afghanistan remains challenging. Decades of protracted conflict, political instability, widespread poverty, and fragile governance systems have collectively weakened health infrastructure, disrupted service delivery, and contributed to persistent inequalities in both access to care and health outcomes across provinces and population groups [7,8]. Rural and hard-to-reach areas, in particular, face compounded challenges to accessing essential services due to geographic isolation, limited transportation networks, shortages of skilled health personnel, and restrictive gender norms.

In response to these longstanding challenges, the Ministry of Public Health introduced the Basic Package of Health Services (BPHS) in 2003 and the Essential Package of Hospital Services (EPHS) in 2005 [9]. These initiatives were designed to expand access to essential healthcare services by decentralizing service delivery and bringing care closer to communities, with a particular focus on women and children in rural and underserved areas. Implementation has largely relied on partnerships with national and international non-governmental organizations (NGOs), supported by substantial donor funding. Over time, these efforts have contributed to notable improvements in key health indicators, including a decline in maternal mortality from 1,600 per 100,000 live births in 2000 to 620 in 2020, a decline in under five mortality from 230 per 1,000 live births in 2004 to 55.0 in 2023, an increase in Diphtheria-Pertussis-Tetanus (DPT3) immunization from 30.1% in 2000 to ~51% in 2023, and an increase in access to basic health services from 9% in 2001 to 87% of the population in 2018 [9–11].

Despite these gains, significant **disparities** in healthcare access persist. Many Afghans continue to face shortages of trained health workers, especially female providers, variability in service quality across provinces, and substantial out-of-pocket costs that lead to financial hardship [12–14]. Ongoing insecurity and recurrent humanitarian crises, including displacement, food insecurity, and natural disasters, exacerbate existing vulnerabilities and disrupt service delivery and access to essential health services [7]. Although national estimates suggest that approximately 90% of the population lives within 2 h of a health facility by available transport [15], these aggregate figures mask substantial subnational variation, with rural, mountainous, and minority-populated regions experiencing poorer access to both primary and secondary care [16].

A critical limitation of existing literature is its reliance on aggregated provincial or national data, which can obscure the depth and heterogeneity of deprivation within provinces, limiting the identification of the most marginalized populations [17]. More granular analyses are therefore needed to characterize multidimensional patterns of health and socioeconomic disadvantage and to inform policies and programs that address both immediate service delivery gaps and the underlying drivers of health disparities [5].

To address this evidence gap, we conducted a comparative analysis of Afghanistan’s provinces using nine complementary indicators encompassing health outcomes, service coverage, access to care, and poverty. Using data from the nationally representative 2022–2023 Multi-Indicator Cluster Survey (MICS) and other national sources, this study quantifies within-country geographic and socioeconomic disparities. The findings are intended to inform evidence-based policy and practice, support more needs-based resource allocation, and guide targeted interventions to reduce health disparities in Afghanistan’s most underserved communities.

## Methods

This study performed a cross-sectional, province-level comparative analysis across all 34 provinces of Afghanistan to assess disparities in health outcomes, service coverage, access to care, and socioeconomic conditions, integrating multiple nationally representative secondary data sources to provide a multidimensional assessment of subnational health disparities.

Three primary sources of data were used: the Afghanistan Multiple Indicator Cluster Survey (MICS) 2022–2023, the joint report by National Statistics and Information Authority (NSIA) and the Ministry of Public Health (MoH) on geographic access to healthcare facilities 2017, and the Afghanistan Multidimensional Poverty Index (MPI) analysis, produced by the National Statistics and Information Authority (NSIA) in partnership with the Oxford Poverty and Human Development Initiative (OPHI) in 2016–2017.

The primary dataset was the Afghanistan MICS 2022–2023, a nationally representative household survey conducted by the United Nations Children’s Fund (UNICEF) in collaboration with the NSIA [11]. Data were collected from September 2022 to February 2023, using a stratified, two-stage cluster sampling design to ensure representativeness at both the national and provincial levels [11]. The MICS provides robust estimates on a wide range of health, nutrition, and development indicators for children, women, and households.

The second data source was the 2017 joint NSIA and MoPH report, which assessed geographic access to healthcare facilities using geospatial analysis [18]. This analysis combined global positioning system (GPS) coordinates of health facilities with population distribution data to estimate the proportion of the population living within defined distances (5 km and 10 km) of health services. For this study, provincial-level estimates of access within 10 km were used as a proxy for physical access to care.

The third source was the Afghanistan Multidimensional Poverty Index (MPI), produced by NSIA in partnership with the Oxford Poverty and Human Development Initiative (OPHI) [19]. The MPI captures deprivation across three domains (health, education, and living standards), and provides province-level estimates of multidimensional poverty [19]. These data were used to assess socioeconomic disparities and their relationship to health disparities.

Although data sources were collected in different years, they represent the most recent nationally representative provincial estimates available for each domain and were combined to provide a comprehensive cross‑sectoral assessment of health and socioeconomic disparities.

Nine indicators were selected for combined analysis, reflecting key aspects of health status, service utilization, and socioeconomic deprivation. These indicators are described in detail in Table 1.

**Table 1.** Indicators, domains, definitions, and data sources used in the composite performance analysis.

No.	Indicator	Domain	Definition	Data source
1	Stunting	Health outcomes	Percentage of children under age 5 who fall below the median height-for-age of the WHO standard: (a) minus two standard deviations (moderate and severe) (b) below minus three standard deviations (severe)	MICS 2022/23 Report
2	Under-five mortality rate	Health outcomes	Probability of dying between birth and the fifth birthday	MICS 2022/23 Report
3	Basic immunization coverage (BCG, third dose of Pentavalent vaccine;third dose of Polio vaccine; first dose of Measles vaccine)	Service coverage	Percentage of children who, at age 12–23 months, had received all basic vaccinations at any time before the survey	MICS 2022/23 Report
4	Antenatal care coverage (ANC4)	Service coverage	Percentage of married women aged 15–49 years with a live birth in the last 2 years who, during the pregnancy of the most recent live birth, were attended at least four times by any provider	MICS 2022/23 Report
5	Skilled birth attendance (SBA)	Service coverage	Percentage of married women aged 15–49 years who had a live birth in the last 2 years and whose most recent delivery was attended by skilled health personnel	MICS 2022/23 Report
6	Postnatal care (PNC)	Service coverage	Percentage of married women aged 15–49 years with a live birth in the last 2 years whose most recent live-born child received a health check while in a facility or at home following delivery, or a postnatal care visit within 2 days after delivery	MICS 2022/23 Report
7	Access to improved drinking water (piped water (into dwelling, compound, yard or plot, to neighbour, public tap/standpipe), tube well/borehole, protected dug well, protected spring, rainwater collection, and packaged or delivered water)	Access to care and living conditions	Percentage of household members using improved sources of drinking water	MICS 2022/23 Report
8	Population access to healthcare within 10 km	Access to care and living conditions	Percentage of households located within 10 kilometers of the nearest health facility, as determined by GPS mapping.	NSIA/MoPH 2017 Report
9	Multidimensional Poverty Index	Socioeconomic status	Percentage of individuals living in households identified as multidimensionally poor. A household is classified as multidimensionally poor if it is deprived in at least 40% of the weighted indicators across five dimensions (health, education, living standards, work, and shocks), following the Afghanistan Multidimensional Poverty Index methodology. The unit of identification is the household, while results are reported for individuals living in those households.	NSIA/OPHI (Oxford Poverty and Human Development Initiative) Report

All indicators were calculated or extracted at the provincial level using standardized definitions from their respective sources.

All data were compiled and managed in Microsoft Excel. Provincial-level values for each indicator were extracted directly from the published reports; no raw survey data were accessed or reanalyzed. These values were then harmonized and organized into a single analytical dataset for composite scoring.

To enable comparison across indicators with different units and scales, a quartile-based scoring approach was applied. This method allows for standardized comparison of provinces across multiple dimensions of health and socioeconomic performance, encompassing health outcomes, service coverage, access, and socioeconomic conditions, and groups provinces into four relative performance categories, allowing consistent comparison despite differences in indicator scales and measurement units. Quartile analysis is particularly appropriate for subnational disparity assessment because it highlights relative disparities within the country, identifies provinces with the widest gaps, and reduces the influence of extreme values.

All analyses were conducted in Microsoft Excel 365 (Microsoft Corporation, Redmond, WA, USA), and R was used for data visualization and figure generation. Provincial-level indicator values were extracted directly from published reports and entered into a single analytical worksheet; no raw survey data were accessed or reanalyzed. To enable comparison across indicators measured on different scales, a provincial tier-based scoring approach was applied. For each indicator, the 34 provinces were ranked according to performance and categorized into four relative performance tiers (Q1–Q4), with Q1 representing the lowest-performing quartile and Q4 the highest-performing quartile. This approach adapts principles of stratified health-inequality assessment used in established equity frameworks to characterize relative differences across subnational administrative units [6,20]. Provinces were ranked from 1 (worst) to 34 (best) using the Excel RANK.EQ function. Tied values received the same rank, with subsequent ranks assigned according to the RANK.EQ procedure. Quartile cut points were determined using the QUARTILE.INC function, and provinces were assigned scores from 1 to 4 according to their position in the distribution. For indicators in which higher values indicated worse outcomes (stunting prevalence, under-five mortality, and multidimensional poverty), the scoring direction was reversed so that higher scores consistently indicated better performance. The composite score for each province was calculated as the unweighted arithmetic mean of the nine indicator scores using the AVERAGE function. All nine indicators were equally weighted because no empirical evidence or policy framework justified assigning greater importance to any individual indicator. R was subsequently used to generate the heat map and provincial map presented in Figures 1 and 2.

**Figure 1.**
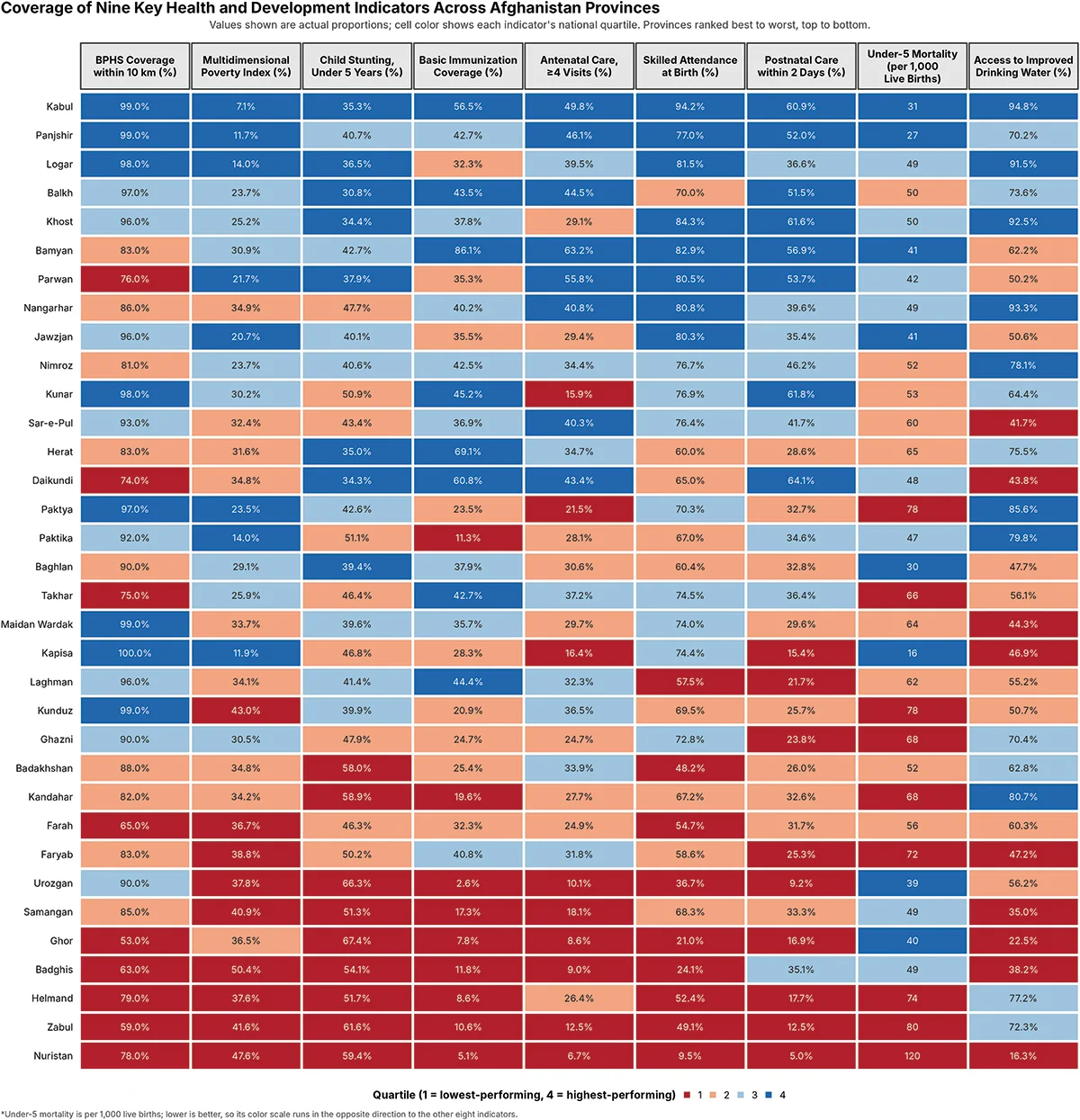
Heatmap of nine health and poverty indicators by province, colored by national quartile.

**Figure 2.**
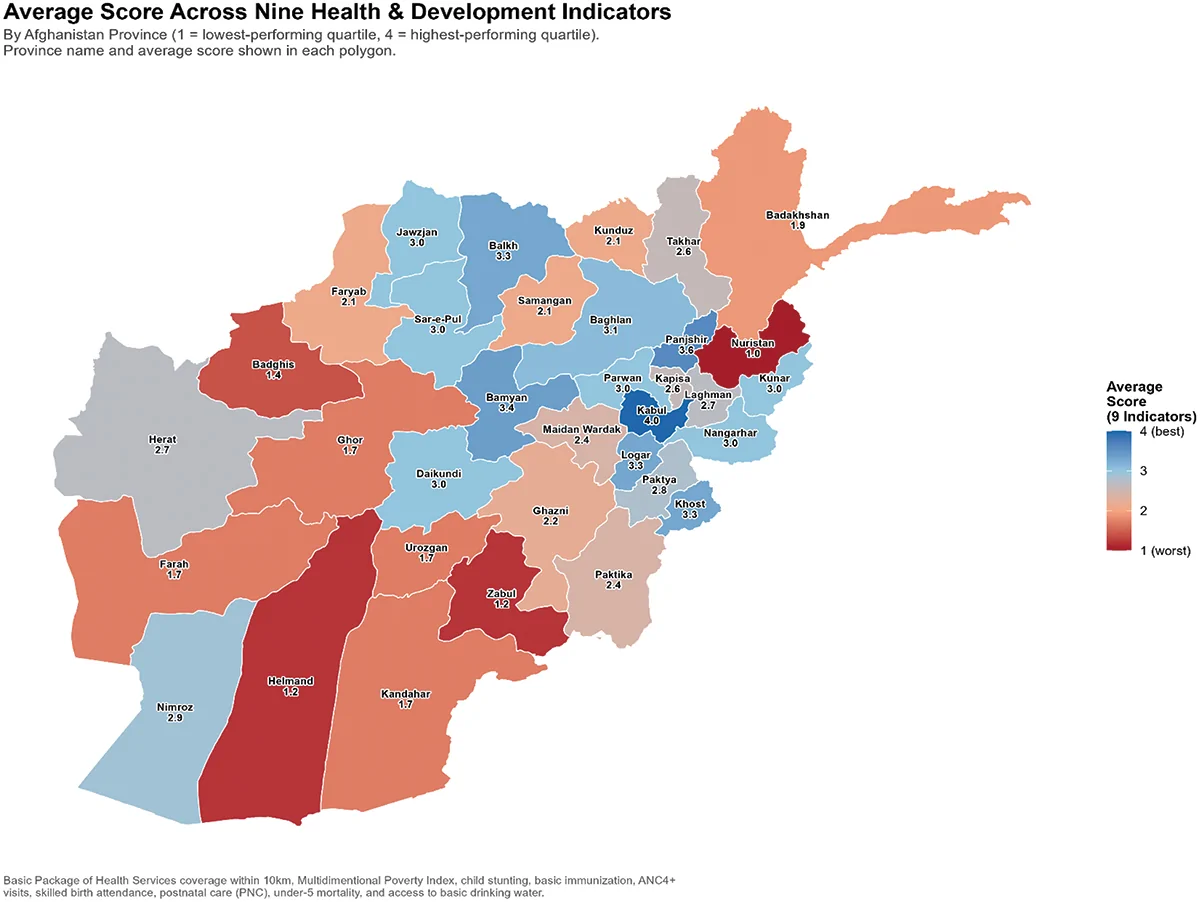
Map of average composite performance score by province.

This study used publicly available, anonymized secondary data and therefore did not require institutional ethics approval.

## Results

Substantial interprovincial variation was observed across all child health indicators, including under-five mortality, stunting, and basic immunization coverage. Under-five mortality ranged from 16 per 1,000 live births in Kapisa to 120 in Nuristan, reflecting pronounced disparities in child survival. Stunting prevalence was highest in Ghor (67.4%), Urozgan (66.3%), and Zabul (61.6%), while comparatively lower, yet still considerable in Balkh (30.8%), Daikundi (34.3%), Herat (35.0%), and Kabul (35.3%). Basic immunization coverage also showed marked variability, ranging from 2.6% in Urozgan and 5.1% in Nuristan to 86.1% in Bamyan and 69.1% in Herat. Overall, these findings highlight substantial geographic disparities in child health outcomes and access to preventive services.

Coverage of maternal health services also varied widely across provinces. Skilled birth attendance ranges from 9.5% in Nuristan to 94.2% in Kabul. High coverage was also observed in Khost (84.3%), Nangarhar (80.8%), Logar (81.5%), and Parwan (80.5%), while substantially lower levels were reported in Ghor (21.0%), Badghis (24.1%), and Urozgan (36.7%). Achievement of at least four antenatal care visits was lowest in Nuristan (6.7%), Ghor (8.6%), and Badghis (9.0%), compared with higher coverage (yet still unacceptably low) in Bamyan (63.2%), Parwan (55.8%), and Kabul (49.8%). Postnatal care (PNC) followed a similar pattern, with very low coverage in Nuristan (5.0%) and Urozgan (9.2%), and higher levels, respectively, in Daikundi (64.1%), Kunar (61.8%), and Kabul (60.9%). These statistics demonstrate substantial disparities in the utilization of maternal health services across provinces, and suboptimal maternal health service usage even in the highest-level provinces.

Access to improved drinking water varied considerably, ranging from 16.3% in Nuristan and 22.5% in Ghor to over 90% in Kabul (94.8%), Nangarhar (93.3%), Khost (92.5%), and Logar (91.5%). Physical access to primary care services, measured as the proportion of the population living within 10 km of a BPHS facility, was generally high but unevenly distributed. Coverage ranged from 53% in Ghor and 59% in Zabul to near universal access in Kapisa (100%) and Kabul (99%), with multiple other provinces exceeding 95%. High geographic access to BPHS facilities was observed in several provinces, although service utilization and health outcomes varied across provinces.

Substantial variation was also observed in socioeconomic conditions. The Multidimensional Poverty Index (MPI) ranged from 7.1% in Kabul to over 50% in Badghis (50.4%), with similarly high levels observed in Nuristan (47.6%) and Zabul (41.6%). Higher MPI values were observed in several provinces that also reported lower levels of health service coverage and less favorable child health indicators. The distribution of all nine indicators across provinces, grouped by national quartile, is shown in Figure 1.

The composite analysis integrating the nine health and development indicators revealed profound multidimensional disparities across provinces. Composite scores ranged from 1.0 (lowest performance) to 4.0 (highest performance), based on quartile rankings across all indicators and representing relative performance across health outcomes, service coverage, healthcare access, and socioeconomic conditions.

The lowest-performing provinces were concentrated in the south, southwest, and western regions. These included Nuristan (1.0, Zabul (1.2), Helmand (1.2), and Badghis (1.4), indicating consistently poor performance across multiple domains). Additional low-performing provinces included Ghor, Farah, Kandahar, and Urozgan (all 1.7), reflecting consistent disadvantages across multiple indicators.

Provinces with intermediate scores ranging from approximately 2.0 to 2.7 demonstrated mixed performance, generally performing well in some domains, while lagging in others. This group was largely concentrated in the northern and northeastern regions, including Badakhshan (1.9), Faryab (2.1), Samangan (2.1), Kunduz (2.1), and Herat (2.7),

The higher-performing provinces were primarily located in the central and northern regions. Provinces with composite scores of 3.0 or above included Daikundi, Parwan, Nangarhar, Sar-e-Pul, Jawzjan, Kunar, Baghlan, and Khost. Kabul (4.0) achieved the highest composite score, followed by Panjshir (3.6), Bamyan (3.4), Balkh (3.3), and Logar (3.3). The geographic distribution of average composite performance scores across all 34 provinces is presented in Figure 2.

## Discussion

This study demonstrates substantial and geographically clustered health disparities across Afghanistan’s provinces. By integrating nine indicators of health outcomes, service coverage, access to care, and multidimensional poverty, the composite analysis shows that deprivation is multidimensional and cumulative, rather than confined to individual indicators. Provinces such as Nuristan, Zabul, Helmand, Badghis, Ghor, and Urozgan consistently ranked among the lowest-performing across multiple domains, whereas Kabul, Panjshir, Bamyan, Logar, and Balkh performed comparatively better. These patterns reveal a clear and persistent subnational disparities that are obscured by national averages.

Although Afghanistan achieved substantial gains in maternal and child health following the introduction of the Basic Package of Health Services (BPHS) and Essential Package of Hospital Services (EPHS, these gains were not distributed evenly across provinces). Recent evidence demonstrates that while national coverage of essential maternal and child health interventions improved over time, provinces such as Nuristan and Urozgan experienced declines in coverage, whereas Daikundi and Nimroz recorded improvements in both coverage and equity [21]. Similarly, prior Afghan evidence reports marked provincial differences in skilled antenatal care, with more than 60% coverage in 20 provinces compared to only 12% in Nuristan [22]. Despite the presence of more than 4,200 primary healthcare facilities nationwide, their distribution remains uneven, leaving nearly one-quarter of the population underserved [5]. Access constraints are substantial in geographically remote regions, including parts of the Central Highlands and western and southern Afghanistan, where underserved populations and service gaps remain concentrated [5].

The present study suggests that geographic isolation does not inevitably translate into poor health and development outcomes. Although remote and mountainous provinces generally performed poorly, important exceptions were observed. Bamyan and Daikundi, despite their geographic isolation, ranked among the highest-performing provinces, while Badakhshan performed comparatively better than Nuristan, despite sharing similar terrain. These findings are consistent with previous research indicating that provincial performance is influenced by factors beyond geography alone, including poverty, conflict intensity, service availability, and access barriers [21,23].

The clustering of disadvantage observed in Nuristan, Zabul, Helmand, Urozgan, Badghis, and Ghor highlights the cumulative nature of deprivation. These provinces consistently experienced poor maternal and child health outcomes, low coverage of essential health services, limited access to basic services, and high multidimensional poverty. Such patterns are consistent with reinforcing prior evidence identifying the southern and western provinces as regions where structural fragility, poverty, and limited service provision are associated with persistent disparities [5,8]. Similar findings have been reported in conflict-affected provinces, where antenatal care, facility delivery, skilled birth attendance, childhood immunization, and treatment-seeking behaviors are significantly lower than in more stable areas [23].

In contrast, provinces such as Kabul, Panjshir, Bamyan, Logar, and Balkh rank among the highest-performing overall. These patterns align with previous evidence indicating that provinces with relatively stronger infrastructure, service availability, and institutional presence tend to have better health outcomes and service coverage [15,16]. Importantly, the relatively strong performance of some geographically isolated provinces suggests that sustained investment and effective health-system organization may mitigate the effects of remoteness.

It is also important to situate these findings within a global frame of reference. Even Afghanistan’s best-performing provinces have not closed the gap with global norms of health service coverage and outcomes. Kabul, the highest-scoring province overall, recorded skilled birth attendance of 94.2%, comparable to the global average of approximately 86% in 2023 [24], yet its antenatal care coverage of only 49.8% remained well below the global average of roughly 70% of pregnant women receiving at least four antenatal visits [25]. Similarly, Kapisa, which recorded the lowest under-five mortality rate of any province (16 per 1,000 live births), still had a rate that was less than half the estimated global average of 37 per 1,000 live births in 2023–2024 [26]. These comparisons indicate that the provinces ranked highest in this analysis are, on several measures, still performing below global benchmarks, underscoring that the disparities documented here reflect both the within-country equity gap and the within-country geographic disparity, as well as a broader gap between Afghanistan and global health standards.

The observed disparities have important implications for Afghanistan’s progress toward Sustainable Development Goals 3 (health and well-being) and 10 (reducing inequality) [27]. National averages may suggest continued progress, yet they may obscure substantial between-province disparities . Without targeted efforts to address these gaps, aggregate improvements are unlikely to translate into more consistent health gains across populations.

These findings are also directly relevant to Universal Health Coverage (UHC) and Afghanistan’s commitments under international human rights frameworks. The right to the highest attainable standard of health requires progressive realization without discrimination. The clustering of deprivation in specific provinces suggests that equal formal availability of services is insufficient; effective access must address barriers related to geography, poverty, and service delivery. Advancing UHC in Afghanistan, therefore, requires targeted investment strategies that prioritize marginalized and geographically isolated populations to fulfill both development commitments and legal obligations related to the right to health [25].

Reducing subnational disparities is central to building resilient health systems capable of withstanding political, economic, and environmental shocks in fragile and conflict-affected settings such as Afghanistan [2]. Persistent disparities weaken overall system performance and limit the ability to respond effectively to ongoing humanitarian, economic, and environmental challenges [2].

A major strength of this study is the use of recent, nationally representative data to provide a multidimensional assessment of subnational health disparities across all 34 provinces of Afghanistan. By integrating nine indicators spanning health outcomes, service coverage, access, and poverty, the study provides a comprehensive assessment of subnational disparities [11,18]. The composite quartile approach enables systematic comparison across provinces and highlights patterns of clustered disadvantage that may be obscured in single-indicator analyses. Importantly, most variables were collected after the political transition, ensuring that the findings reflect Afghanistan’s current governance and service delivery context. However, several limitations should be noted. First, the analysis relies on secondary data sources collected in different years, which may introduce temporal inconsistencies. Second, important dimensions of population health, such as indicators for non-communicable diseases and injuries, were not included due to a lack of comparable provincial data. Third, provincial-level averages may mask substantial within-province heterogeneity, particularly in geographically diverse or hard-to-reach areas. More granular, district-level analyses would likely reveal additional layers of inequality. Fourth, all indicators were assigned equal weight in the composite index because no empirical evidence supported differential weighting; alternative weighting approaches should be explored in future studies. Finally, provinces were grouped into quartiles rather than finer categories such as quintiles or deciles. In contrast, quartiles were chosen for interpretability, given the modest number of provinces (*n* = 34) and the composite nature of the index; finer groupings could reveal additional within-group heterogeneity and should be considered in future analyses.

The clustering of multidimensional deprivation identified in this study highlights the need for geographically targeted responses that address the different patterns of disadvantage across provinces. Rather than applying the same approach everywhere, priorities should be guided by the specific gaps observed in each province. Nuristan, which had the lowest composite score, also had particularly low coverage of antenatal care, skilled birth attendance, and postnatal care, as well as limited access to improved drinking water. These findings point to the importance of strengthening community-based maternal and newborn health services, increasing the availability of female health workers, expanding outreach services, and improving access to safe water. In Ghor, high stunting occurred alongside low maternal health service coverage, limited access to improved drinking water, and the lowest geographic access to health facilities, suggesting that nutrition, maternal health, water and sanitation, and access to health services should be addressed together. Urozgan had particularly low immunization and postnatal care coverage as well as high stunting, highlighting childhood immunization, maternal and postnatal outreach, and nutrition as important priorities. In Zabul, limited geographic access to health facilities, high stunting, and low coverage of preventive services point to the need for stronger outreach and community-based maternal and child health services. Badghis combined high multidimensional poverty with very low antenatal care and skilled birth attendance, suggesting that efforts to improve maternal health services should be accompanied by broader measures addressing socioeconomic disadvantage. Other lower-performing provinces, including Helmand, Kandahar, Farah, and Paktika, would similarly benefit from responses tailored to their particular gaps in service coverage, access, and socioeconomic conditions.

Across lower-performing provinces, expanding community-based service delivery models [28], including mobile health teams, community midwives, and local health workers, may help reach remote and underserved populations. Addressing social and gender-related barriers to care also remains important, particularly through the recruitment and retention of female health workers and broader efforts to improve women’s access to education and health services [29]. The overlap between multidimensional poverty and poorer health indicators also points to the importance of coordinated approaches that address health alongside water and sanitation, nutrition, education, food security, and livelihoods. Strengthening subnational data systems will also be important for identifying remaining disparities, guiding resource allocation, and targeting interventions more precisely at provincial and district levels.

## Conclusion

This study documents severe and persistent subnational disparities in maternal and child health outcomes, service access, and multidimensional poverty across Afghanistan. Provinces such as Nuristan and several southern and western provinces experience compounded disadvantage across multiple domains, while central and northern provinces demonstrate comparatively better outcomes. These findings underscore the need for sustained, targeted strategies that address both immediate service delivery gaps and the broader structural determinants of health. Addressing these health disparities in Afghanistan will require long-term commitment, targeted investment, and policies that prioritize the most underserved populations.

## Data Availability

The datasets analyzed during this study are publicly available from the following sources: the Afghanistan Multiple Indicator Cluster Survey (MICS) 2022–2023, UNICEF (https://mics.unicef.org/country-profiles/afghanistan/3746); the National Statistics and Information Authority (NSIA) of Afghanistan (https://nsia.gov.af/home), which produced the 2017 joint NSIA/Ministry of Public Health geographic access-to-care report; and the Afghanistan Multidimensional Poverty Index, Oxford Poverty and Human Development Initiative (OPHI) (https://ophi.org.uk/national-mpi-directory/afghanistan-mpi).
